# Introgression of two chromosome regions for leaf photosynthesis from an *indica* rice into the genetic background of a *japonica* rice

**DOI:** 10.1093/jxb/eru047

**Published:** 2014-03-03

**Authors:** Shunsuke Adachi, Leticia Z. Baptista, Tomohiro Sueyoshi, Kazumasa Murata, Toshio Yamamoto, Takeshi Ebitani, Taiichiro Ookawa, Tadashi Hirasawa

**Affiliations:** ^1^Graduate School of Agriculture, Tokyo University of Agriculture and Technology, Fuchu 183–8509, Japan; ^2^Japan Science and Technology Agency, Precursory Research for Embryonic Science and Technology, Kawaguchi 332-0012, Japan; ^3^National Institute of Agrobiological Sciences, Tsukuba 305–8602, Japan; ^4^Toyama Prefectural Agricultural, Forestry and Fisheries Research Center, Toyama 939–8153, Japan

**Keywords:** Hydraulic conductance, leaf nitrogen content, *Oryza sativa*, photosynthesis, quantitative trait locus, stomatal conductance.

## Abstract

Increases in rates of individual leaf photosynthesis (*P*_n_) are critical for future increases of rice yields. A previous study, using introgression lines derived from a cross between indica cultivar Habataki, with one of the highest recorded values of *P*_n_, and the Japanese elite cultivar Koshihikari, identified four QTLs (qCAR4, qCAR5, qCAR8, and qCAR11) that affect *P*_n_. The present study examined the combined effect of qCAR4 and qCAR8 on *P*_n_ in the genetic background of Koshihikari. The pyramided near-isogenic line NIL(qCAR4+qCAR8) showed higher *P*_n_ than both NIL(qCAR4) and NIL(qCAR8), equivalent to that of Habataki despite being due to only two out of the four QTLs. The high *P*_n_ of NIL(qCAR4+qCAR8) may be attributable to the high leaf nitrogen content, which may have been inherited from NIL(qCAR4), to the large hydraulic conductance due to the large root surface area from NIL(qCAR4), and to the high hydraulic conductivity from NIL(qCAR8). It might be also attributable to high mesophyll conductance, which may have been inherited from NIL(qCAR4). The induction of mesophyll conductance and the high leaf nitrogen content and high hydraulic conductivity could not be explained in isolation from the Koshihikari background. These results suggest that QTL pyramiding is a useful approach in rice breeding aimed at increasing *P*_n_.

## Introduction

Increasing the rates of leaf photosynthesis (*P*_n_) should increase the yield potential of rice (*Oryza sativa* L.), since *P*_n_ affects dry matter production via photosynthesis within the canopy (Long *et al.*, 2006; Murchie *et al.*, 2009). The use of natural genetic variation in photosynthesis within species can be an effective strategy for crop improvement (Flood *et al.*, 2011). Wide variations in *P*_n_ among rice cultivars have been shown in a number of studies (Murata, 1961; Takano and Tsunoda, 1971; Cook and Evans, 1983; Yeo *et al.*, 1994; Osada, 1995; Xu *et al.*, 1997; Masumoto *et al.*, 2004; Kanemura *et al.*, 2007; Jahn *et al.*, 2011). However, most of the natural genetic resources have yet to be tapped.

Quantitative genetics is useful in assessing the genetic factors underlying the variation in photosynthesis and in designing breeding programmes (Flood *et al.*, 2011). The complete genome sequence of rice and many DNA markers are already available (IRGSP, 2005). Several advanced populations, including backcrossed inbred lines and chromosome segment substitution lines, have been developed to facilitate the genetic analysis of rice (Yamamoto *et al.*, 2009; Fukuoka *et al.*, 2010). Recent improvements in the quantitation of photosynthesis have reduced measurement times while maintaining accuracy in the field (Long and Bernacchi, 2003). These advances facilitate the identification of quantitative trait loci (QTLs) and isolation of the underlying genes. In recent studies, several QTLs for *P*_n_ have been identified in rice (Teng *et al.*, 2004; Hu *et al.*, 2009; Takai *et al.*, 2010; Gu *et al.*, 2012), and one gene has been isolated (Takai *et al.*, 2013).

The combination of multiple QTLs—QTL pyramiding—offers a straightforward and useful way for improving target traits in rice (Wang *et al.*, 2012). To evaluate the precise effects of the combination of QTLs, it is necessary to develop near-isogenic lines (NILs), which carry a single target QTL in a unique genetic background to eliminate background noise, and to cross these NILs (Ashikari and Matsuoka, 2006; Hospital, 2009). However, there has been limited effort so far to develop NILs for rice *P*_n_ (Gu *et al.*, 2012) and no attempt to evaluate the effect of pyramiding QTLs on *P*_n_.

It is widely acknowledged that *P*_n_ is closely related to leaf nitrogen content (LNC) in rice, because large amounts of N are invested in ribulose-1,5-bisphosphate carboxylase/oxygenase (Rubisco), the primary CO_2_-fixation enzyme (Cook and Evans, 1983; Makino *et al.*, 1992). Wide varietal differences in LNC have been observed even at the same rate of N application (Kanemura *et al.*, 2007; Hirasawa *et al.*, 2010). *P*_n_ is also affected by the diffusion of CO_2_ from the atmosphere to the chloroplasts. Varietal differences in stomatal conductance (*g*_s_) have been observed even at a small vapour pressure deficit (Ohsumi *et al.*, 2008; Hirasawa *et al.*, 2010). Since *g*_s_ decreases as leaf water potential decreases, the hydraulic conductance from roots to leaves (*C*_p_), which controls the water balance in plants, would affect the value of *g*_s_ (Hirasawa *et al.*, 1989; Hirasawa and Ishihara, 1992; Brodribb *et al.*, 2007). Mesophyll conductance (*g*_m_), with respect to the diffusion of CO_2_ from the intercellular airspace to the chloroplasts, might also be important to improving rice *P*_n_ (Makino, 2011). Recent studies report genetic differences in *g*_m_ among *Oryza* species (Scafaro *et al.*, 2011) and rice lines (Adachi *et al.*, 2013). In addition to Rubisco content, LNC is associated with also *g*_s_ and *g*_m_ (Makino *et al.*, 1988; Ishihara, 1995; Li *et al.*, 2009). To clarify factors underlying the differences in *P*_n_ among rice cultivars and lines, the influences of differences in LNC should be considered carefully.

The *P*_n_ of young, newly expanded leaves ranges between ~20 and ~30 μmol CO_2_ m^–2^ s^–1^ among rice cultivars at an ambient CO_2_ concentration of 370–400 μmol mol^–1^ under light-saturating and unstressed conditions (Kanemura *et al.*, 2007; Hirasawa *et al.*, 2010; Jahn *et al.*, 2011). The high-yielding *indica* cultivar Habataki has one of the highest recorded rates of *P*_n_ among rice cultivars, at 30–33 μmol m^–2^ s^–1^ (Asanuma *et al.*, 2008). In contrast, Koshihikari, the most popular cultivar in Japan, has a relatively low *P*_n_ of 25–28 μmol m^–2^ s^–1^.

In previous studies using introgression lines derived from a cross between Koshihikari and Habataki, this study group identified four QTLs (*qCAR4* on chromosome 4, *qCAR5* on chromosome 5, *qCAR8* on chromosome 8, and *qCAR11* on chromosome 11), Habataki alleles of which increased *P*_n_ (Sueyoshi *et al.*, 2009; Adachi *et al.*, 2011). The present work developed a pyramided line, NIL(*qCAR4+qCAR8*), by crossing NIL(*qCAR4*) and NIL(*qCAR8*), each of which has a single chromosome segment from Habataki substituted in the genetic background of Koshihikari, and quantified the effects on the rate of leaf photosynthesis and on processes related to photosynthesis.

## Materials and methods

### Plant materials

**Fig. 1.**
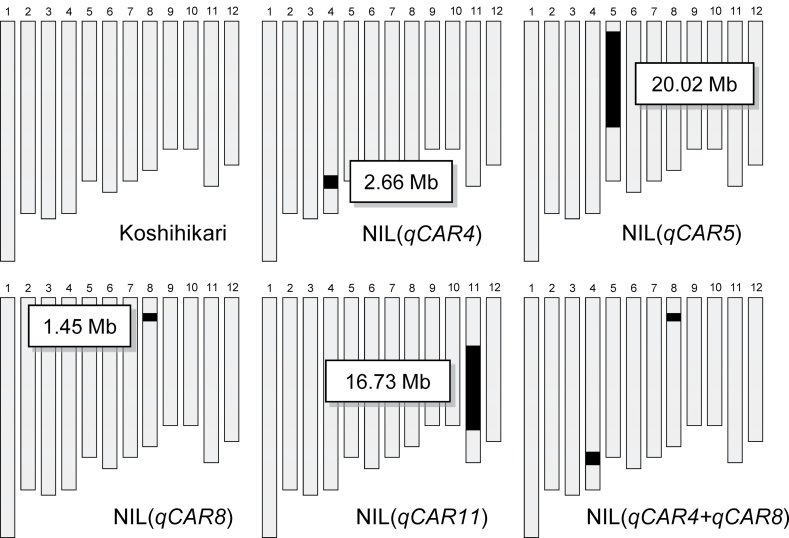
Graphical genotypes of Koshihikari and the quantitative trait loci near-isogenic lines. Black bars indicate regions homozygous for Habataki alleles. Values in boxes indicate lengths of substituted regions.

Four QTL-NILs carrying a single chromosome segment from Habataki in the genetic background of Koshihikari were developed (Fig. 1; Sueyoshi *et al.*, 2009; Adachi *et al.*, 2011). NIL(*qCAR4*), which carries a chromosome segment from Habataki on the long arm of chromosome 4, was crossed with NIL(*qCAR8*), with a segment from the short arm of chromosome 8, and plants homozygous for Habataki in both regions were selected, to create the line NIL(*qCAR4+qCAR8*). F_2_ progeny were used in field experiments and F_3_ progeny were used in pot experiments.

### Cultivation of rice plants

Plants were grown at the university farm (35° 40′ N 139° 28′ E). In the field experiment, seedlings at the fourth-leaf stage were transplanted into the paddy field (alluvial clay loam) at a density of 22.2 hills m^–2^ (30×15cm), with one plant per hill. As a basal dressing, manure was applied at 15 t ha^–1^ and inorganic fertilizer was applied at 30kg N, 60kg P_2_O_5_, and 60kg K_2_O ha^–1^. One-third of the total N was applied as nitrogen sulphate, one-third as LP-50 elution-controlled urea (Chisso Asahi Fertilizer, Tokyo, Japan), and one-third as LPS-100 elution-controlled urea. No topdressing was applied.

In the pot experiment, plants were grown outdoors in 12-l pots filled with a 1:1 (v/v) mixture of paddy soil and upland soil (diluvial volcanic ash) at a density of three hills per pot, three plants per hill. Basal fertilizer was applied at 1.0g N, 1.0g P_2_O_5_, and 1.0g K_2_O per pot, and additional N was applied at 0.5g per pot at booting stage when the flag leaves had fully expanded. To examine the relationship between *P*_n_ and LNC, different amounts of N were applied to Koshihikari and Habataki at booting stage: at 0.25, 0.5, 1.0 or 2.0g to Koshihikari and 0, 0.25 or 0.5g to Habataki.

Plants were also grown in 3-l pots in a growth chamber (Koitotron, Koito Manufacturing, Tokyo, Japan) under a 12/12 light/dark cycle (28/23 °C, relative humidity 60/80%, and a photosynthetic photon flux density (PPFD) at the top of the canopy of 1000 μmol m^–2^ s^–1^). Basal fertilizer was applied at 0.5g N, 0.5g P_2_O_5_, and 0.5g K_2_O per pot. No topdressing was applied.

### Gas exchange measurements

Leaf gas exchange was measured with a portable gas-exchange system (LI-6400, LI-COR, Lincoln, NE, USA); flag leaves were measured at the full heading stage. The CO_2_ assimilation rate (*A*_370_) and stomatal conductance (*g*_s_) were measured at an ambient CO_2_ concentration of 370 μmol mol^–1^, a PPFD of 2000 μmol m^–2^ s^–1^, a leaf-to-air vapour pressure difference of 1.3–1.6 kPa, and an air temperature of 30 °C. The CO_2_ assimilation rate at an intercellular CO_2_ concentration (*C*_i_) of 280 μmol mol^–1^ (*A*_280_) was also measured by changing the ambient CO_2_ concentration. Plants were examined from 08:30 to 11:00, when the photosynthetic rate was close to the daily maximum (Hirasawa and Ishihara, 1992; Ishihara, 1995). Measurements were made at the full heading stage. The measurement for each leaf was conducted once a day and was repeated on day 2 or 3 and the mean of measurements calculated. Between five and seven leaves were used for each replicate.

### Determination of nitrogen content and dry matter weight

The leaves were collected immediately after completion of the gas exchange measurements and were stored at –80 °C. The area of an 80-mm-long segment cut from the centre of the leaf was measured with a leaf area meter (AAM-9; Hayashi Denko, Tokyo, Japan). The segments were dried at 80 °C for 24h to determine the nitrogen content with a CN analyser (MT700 Mark II, Yanako, Kyoto, Japan) and the dry matter weight.

### Determination of hydraulic conductance and conductivity

The hydraulic conductance of the plants grown in 3-l pots, from the soil through the roots to the leaves (*C*_p_, 10^–8^ m^3^ s^–1^ MPa^–1^) was calculated as *U*_w_/(Ψ_s_–Ψ_l_) (Hirasawa and Ishihara, 1991), where *U*_w_ (10^–8^ m^3^ s^–1^) is the water uptake rate of the whole plant, Ψ_s_ (MPa) is the water potential of the soil immediately outside the root, and Ψ_l_ (MPa) is the average water potential of the uppermost three leaves. Since plants were grown under submerged conditions and the water potential of the soil solution was high compared with Ψ_l_ and was kept constant, Ψ_s_ could be regarded as 0. Measurements were made in a controlled-environment chamber (air temperature 28 °C, air vapour pressure deficit 1.5 kPa, PPFD at the top leaves 1000 μmol m^–2^ s^–1^). *U*_w_ was determined from the rate of weight loss of the pot over 20min after a steady state had been reached. To prevent evaporation from the surface of the pot, the top was covered with polystyrene foam and the gap between the foam and the stem was sealed with oil clay. After measurement of *U*_w_, Ψ_l_ of the uppermost three leaves was measured in a pressure chamber (model 3005, Soil Moisture Equipment, Santa Barbara, CA, USA). Measurements were conducted under the steady-state condition where the transpiration rate is equal to *U*_w_. It is reported that the transpiration rate and *g*_s_ do not influence hydraulic conductance when the transpiration rate is high (Fiscus, 1975; Hirasawa and Ishihara, 1991; Stiller *et al.*, 2003). The *U*_w_ per leaf area was sufficiently high (>2.0 mmol m^–2^ s^–1^) to eliminate the effect of the difference in water uptake rate on *C*_p_. After roots had been washed gently in water, the root surface area of the total root system (*S*_r_) was measured with an image analyser (Win-Rhizo REG V 2004 b, Regent, PQ, Canada). For comparing root hydraulic conductivity, the hydraulic conductivity of a plant (*L*_p_, 10^–8^ m s^–1^ MPa^–1^), defined as hydraulic conductance per root surface area (Steudle and Peterson, 1998).

## Results

### Leaf photosynthesis

**Fig. 2.**
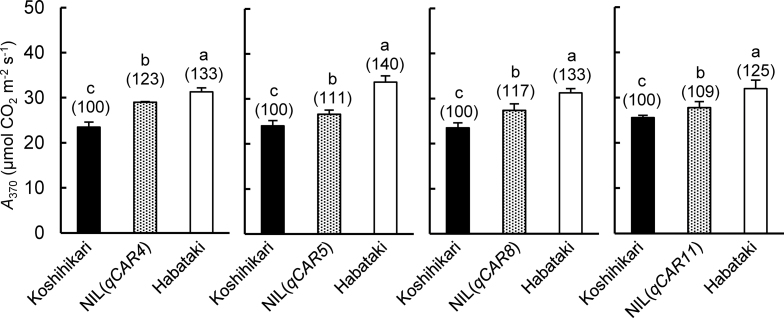
Comparison of CO_2_ assimilation rates at a photosynthetic photon flux density of 2000 μmol m^–1^ s^–1^ and an ambient CO_2_ concentration of 370 μmol mol^–1^ (*A*_370_) of Koshihikari, Habataki, and the quantitative trait loci near-isogenic lines in the paddy field. Measurements for each panels were conducted in different field plots. Data are mean±SD (*n*=3–5). Values above bars are percentages relative to Koshihikari. Different letters above bars indicate significant differences (*P*<0.05, Tukey’s test).

The CO_2_ assimilation rate at an ambient CO_2_ concentration of 370 μmol mol^–1^ (*A*_370_) in the four NILs were significantly higher than that in Koshihikari—by 23% in NIL(*qCAR4*), 11% in NIL(*qCAR5*), 17% in NIL(*qCAR8*), and 9% in NIL(*qCAR11*)—and significantly smaller than that in Habataki—7, 21, 12, and 13%, respectively (Fig. 2). In the field experiment, *A*_370_ in NIL(*qCAR4+qCAR8*) was higher than in both NIL(*qCAR4*) and NIL(*qCAR8*) (Table 1), indicating that the combination of the two QTLs additively increased leaf photosynthesis. Interestingly, *A*_370_ in NIL(*qCAR4+qCAR8*) was comparable to that in Habataki despite being due to only two out of the four QTLs. Similar results were obtained in the pot experiments.

**Table 1.** CO_2_ assimilation rate at a PPFD of 2000 μmol m^–1^ s^–1^ and an ambient CO_2_ concentration of 370 μmol mol^–1^, leaf nitrogen content, dry matter weight per leaf area, and stomatal conductance of plants grown in the field and in 12-l potsValues are means±SD (*n*=3–6). Different superscript letters indicate significant differences between rice lines (*P*<0.05, Tukey’s test). Values in parentheses are percentages relative to Koshihikari. *A*_370_, CO_2_ assimilation rate at an ambient CO_2_ concentration of 370 μmol mol^-1^; *g*_s_, stomatal conductance; LMA, dry matter weight per leaf area; LNC, leaf nitrogen content.

		*A*_370_ (μmol CO_2_ m^–2^ s^–1^)	LNC (g m^–2^)	LMA (g m^–2^)	*g*_s_ (mol H_2_O m^–2^ s^–1^)
Paddy field	Koshihikari	23.5±1.1^c^	1.46±0.15^b^	58.4±4.1^b^	0.66±0.06^c^
	NIL(*qCAR4*)	29.0±0.1 ^a,b^ (123)	1.85±0.06^a^ (127)	66.7±1.8^a^ (114)	0.82±0.07 ^b,c^ (124)
	NIL(*qCAR8*)	27.4±1.4^b^ (117)	1.59±0.15 ^a,b^ (109)	59.1±1.2^b^ (101)	0.85±0.06 ^b,c^ (129)
	NIL(*qCAR4+qCAR8)*	31.0±1.2^a^ (132)	1.81±0.17^a^ (124)	65.2±0.4^a^ (112)	1.01±0.05^b^ (152)
	Habataki	31.3±0.9^a^ (133)	1.85±0.07^a^ (126)	57.6±0.5^b^ (99)	1.82±0.12^a^ (274)
Pots	Koshihikari	22.5±1.5^d^	1.35±0.06^d^	55.8±1.1^b^	0.55±0.05^d^
	NIL(*qCAR4*)	28.8±0.8^b^ (129)	1.67±0.11 ^b,c^ (124)	63.6±2.8^a^ (115)	0.82±0.03^b^ (151)
	NIL(*qCAR8*)	26.1±0.8^c^ (117)	1.54±0.06^c^ (115)	57.4±1.1^b^ (104)	0.70±0.02^c^ (128)
	NIL(*qCAR4+qCAR8)*	31.0±0.5^a^ (139)	1.91±0.12^a^ (142)	64.2±3.1^a^ (116)	0.87±0.04^b^ (159)
	Habataki	31.5±1.8^a^ (141)	1.80±0.06 ^a,b^ (133)	53.8±1.1^b^ (97)	1.81±0.15^a^ (331)

### Leaf nitrogen content

In the field experiment, LNC was significantly higher in NIL(*qCAR4*) (by 27%) than in Koshihikari, but LNC in NIL(*qCAR8*) was similar to that in Koshihikari (Table 1). LNC in NIL(*qCAR4+qCAR8*) was higher than that in Koshihikari (by 24%) and similar to those in NIL(*qCAR4*) and Habataki. In the pot experiment, not only NIL(*qCAR4*) but also NIL(*qCAR8*) showed higher LNC than Koshihikari. LNC in NIL(*qCAR4*+*qCAR8*) was even higher than that in NIL(*qCAR4*) (Table 1).

### Dry matter weight per leaf area

In the field experiment, dry matter weight per leaf area (LMA) in Habataki was similar to that in Koshihikari (Table 1). LMA was significantly higher in NIL(*qCAR4*) (by 14%) than in Koshihikari, but LMA in NIL(*qCAR8*) was similar to that in Koshihikari. LMA in NIL(*qCAR4+qCAR8*) was higher than that in Koshihikari (by 12%) and similar to that in NIL(*qCAR4*). Similar results were obtained in the pot experiment.

### Stomatal conductance and hydraulic conductance

In the field experiment, *g*_s_ was 24% higher in NIL(*qCAR4*) and 29% higher in NIL(*qCAR8*) than in Koshihikari (Table 1). It was 52% higher in NIL(*qCAR4+qCAR8*) than in Koshihikari and was higher than those in both NIL(*qCAR4*) and NIL(*qCAR8*), but still lower than that in Habataki. Similar results were obtained in the pot experiment.

**Fig. 3.**
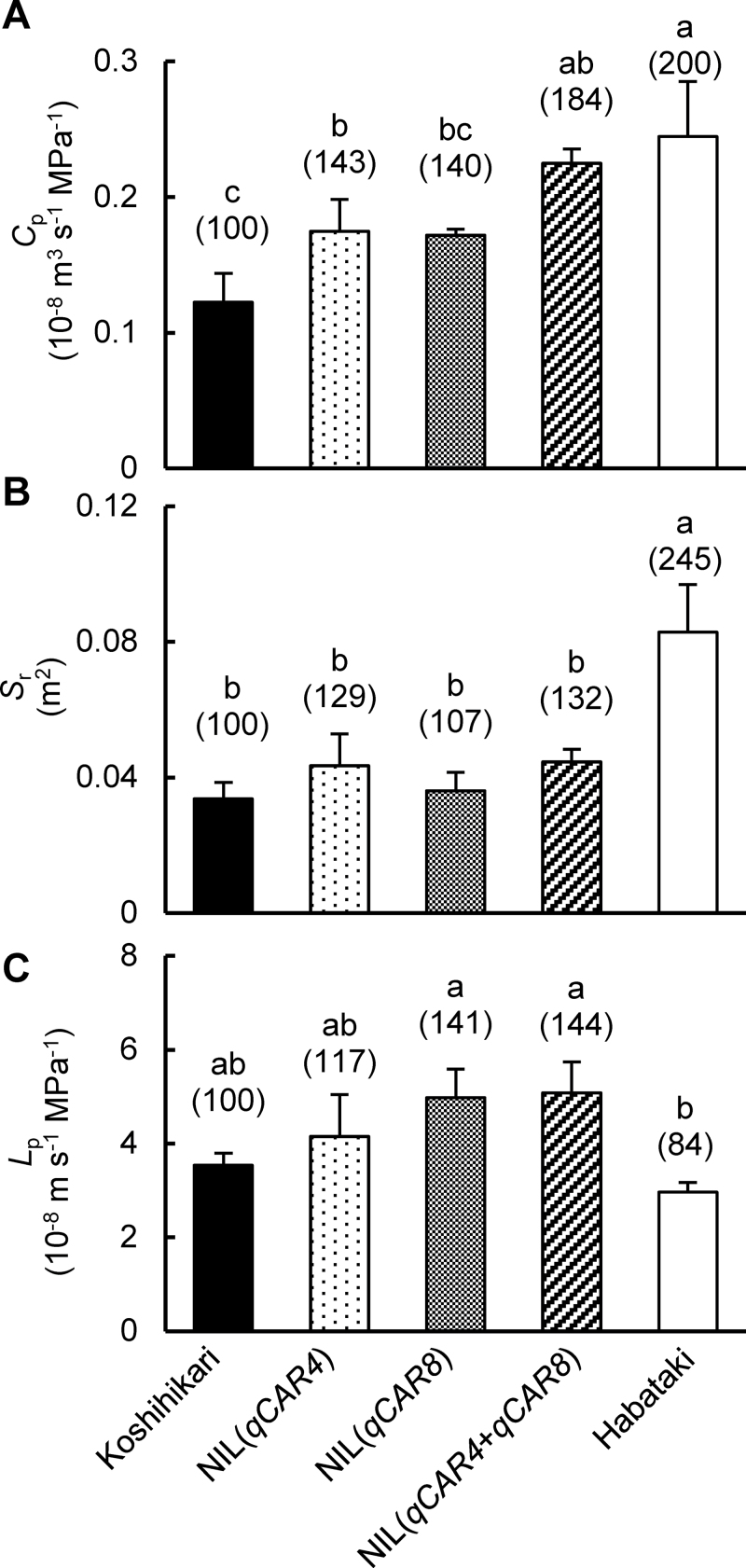
(A) Hydraulic conductance from roots to leaves (*C*_p_), (B) root surface area (*S*_r_), and (C) hydraulic conductivity (*L*_p_) of plants grown in 3-l pots. *C*_p_ and *S*_r_ are expressed on a per stem basis. Values are mean±SD (*n*=4). Values above bars are percentages relative to Koshihikari. Different letters above bars indicate significant differences (*P*<0.05, Tukey’s test).

*C*_p_ was significantly higher in Habataki than in Koshihikari (Fig. 3A). It was significantly higher in NIL(*qCAR4*) (by 43%) and NIL(*qCAR8*) (by 40%) than in Koshihikari. It was 84% higher in NIL(*qCAR4*+*qCAR8*) than in Koshihikari, and was higher in NIL(*qCAR4*+*qCAR8*) than in NIL(*qCAR4*) (*P*=0.07) and NIL(*qCAR8*) (*P*=0.05), but still lower than in Habataki.

*C*_p_ is related to *S*_r_ and *L*_p_ (Steudle and Peterson, 1998). The high value of *S*_r_ in Habataki contributed to the high *C*_p_ in Habataki (Fig. 3A, B). *S*_r_ in NIL(*qCAR8*) was similar to that in Koshihikari, while that in NIL(*qCAR4*) was higher (although not significantly) than that in Koshihikari (Fig. 3B). *S*_r_ in NIL(*qCAR4*+*qCAR8*) was higher than that in Koshihikari and similar to that in NIL(*qCAR4*). *L*_p_ in Habataki was lower than that in Koshihikari (Fig. 3C). *L*_p_ in NIL(*qCAR4*) was similar to that in Koshihikari, while *L*_p_ in NIL(*qCAR8*) was higher (although not significantly) than that in Koshihikari. *L*_p_ in NIL(*qCAR4*+*qCAR8*) was higher than that in Koshihikari and similar to that in NIL(*qCAR8*). These results indicate that the elevated *C*_p_ in NIL(*qCAR4*+*qCAR8*) resulted from simultaneous increases of *S*_r_, which may have been inherited from NIL(*qCAR4*), and *L*_p_, from NIL(*qCAR8*).

### Relationship between *P*_n_ and LNC

**Fig. 4.**
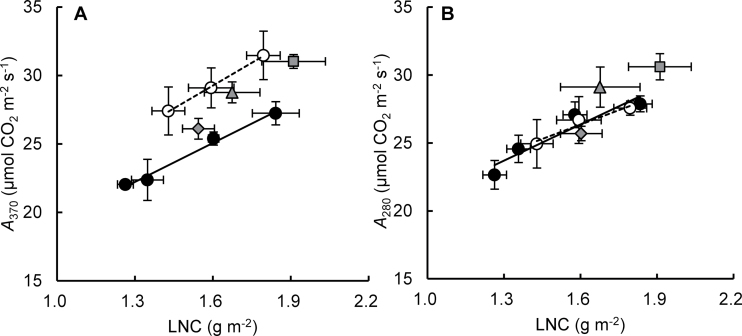
Relationships between leaf N content (LNC) and (A) CO_2_ assimilation rate at an ambient CO_2_ concentration of 370 μmol mol^–1^ (*A*_370_) and (B) an intercellular CO_2_ concentration of 280 μmol mol^–1^ (*A*_280_) in flag leaves of Koshihikari (filled circles), Habataki (open circles), NIL(*qCAR4*) (triangles), NIL(*qCAR8*) (diamonds), and NIL(*qCAR4*+*qCAR8*) (squares) grown in 12-l pots. The solid and broken lines indicate the regression lines for Koshihikari and Habataki, respectively. Values are mean±SD (*n*=4–6).

*A*_370_ was always higher in Habataki than in Koshihikari at a given LNC and it increased with increases in LNC in both cultivars (Fig. 4A). *A*_370_ was also higher in NIL(*qCAR4*), NIL(*qCAR8*), and NIL(*qCAR4*+*qCAR8*) than in Koshihikari at the same LNC. It is possible to estimate photosynthetic activity without considering the effect of *g*_s_ by measuring *P*_n_ at identical *C*_i_ (von Caemmerer and Farquhar, 1981). There was no difference in *A*_280_ between Koshihikari and Habataki at all levels of LNC examined (Fig. 4B). This indicates that the difference in *A*_370_ was entirely due to the differences in LNC and *g*_s_ between Koshihikari and Habataki (Supplementary Fig. S1 available at *JXB* online). *A*_280_ in NIL(*qCAR8*) was similar to that in Koshihikari at the same LNC, while *A*_280_ in NIL(*qCAR4*) and NIL(*qCAR4*+*qCAR8*) was higher than that in Koshihikari at given values of LNC. This indicates that factors other than LNC and *g*_s_ are related to the high *A*_370_ in both NIL(*qCAR4*) and NIL(*qCAR4*+*qCAR8*).

## Discussion

Pyramiding of *qCAR4* and *qCAR8*, which are QTLs for leaf photosynthesis, additively increased *P*_n_ in rice. Similar approaches have yielded additive increases in number of grains (Ashikari *et al.*, 2005; Ando *et al.*, 2008), grain yield (Ohsumi *et al.*, 2011; Wang *et al.*, 2012), grain quality (Wang *et al.*, 2012), plant height (Wang *et al.*, 2012), and heading date (Shibaya *et al.*, 2011; Wang *et al.*, 2012). This is the first report to show that QTL pyramiding is effective at improving rice leaf photosynthesis.

Habataki has one of the highest recorded values of *P*_n_ among rice cultivars, and at least four QTLs for leaf photosynthesis are associated with the difference in *P*_n_ between Koshihikari and Habataki (Asanuma *et al.*, 2008; Sueyoshi *et al.*, 2009; Adachi *et al.*, 2011). Because *A*_370_ in NIL(*qCAR4*+*qCAR8*), with two of the four QTLs, was the same as in Habataki, with all four QTLs (Table 1), the possible reasons for the high *A*_370_ need to be discussed from both genetic and physiological viewpoints.

### Possible reasons for the high rate of leaf photosynthesis in the pyramided line

LNC strongly affects *P*_n_ because it is closely related to the content of Rubisco (Makino *et al.*, 1992). A difference in Rubisco content is a key factor in varietal differences in the capacity for leaf photosynthesis in rice (Hubbart *et al.*, 2007; Hirasawa *et al.*, 2010). In addition, the diffusion of CO_2_ from the atmosphere to the chloroplasts, which is regulated by *g*_s_ and *g*_m_, is another important determinant (Ohsumi *et al.*, 2008; Hirasawa *et al.*, 2010; Makino, 2011). The high *P*_n_ of Habataki might result from the high LNC, due to its elevated capacity for N accumulation, and from the high *g*_s_, due to the large hydraulic conductance of plants, in turn due to the large root surface area compared with Koshihikari (Adachi *et al.*, 2011).

In the field experiment, the higher LNC in NIL(*qCAR4*+*qCAR8*) would have contributed to the higher *A*_370_ than that in Koshihikari (Table 1). The high LNC may have been inherited from NIL(*qCAR4*), because the values of LNC in these two NILs were similar and reached the level of Habataki. In a preliminary study, NIL(*qCAR5*) and NIL(*qCAR11*) also showed higher LNC than Koshihikari (data not shown). This result suggests that at least three QTLs are involved in the difference in LNC between Koshihikari and Habataki. The large increase in LNC in NIL(*qCAR4*+*qCAR8*) and NIL(*qCAR4*) cannot be explained in isolation from the Koshihikari background: that is, there might be other QTLs, the Koshihikari alleles of which increase LNC, or genetic interactions between *qCAR4* and other unknown QTLs. The detailed genetic mechanisms should be examined in future research. In the pot experiment, LNC in NIL(*qCAR8*) was also higher than that in Koshihikari. This suggests that *qCAR8* is also associated with LNC under some growing conditions. The interaction between genes related to photosynthesis and the environment would be another subject to study.

The high *g*_s_ in NIL(*qCAR4*+*qCAR8*) may also have contributed to the higher *A*_370_ than in Koshihikari (Table 1). *g*_s_ was additively increased by the combination of *qCAR4* and *qCAR8*. Although *g*_s_ in Habataki was significantly higher than that in NIL(*qCAR4*+*qCAR8*), a greater *g*_s_ might not increase photosynthesis in rice further (Hirasawa *et al.*, 1988), as Habataki had a much greater *g*_s_ but no greater *A*_370_. The critical water potential for stomatal closure is very much higher in rice than in other crop plants (Hirasawa, 1999). The value of *g*_s_ in Koshihikari was already decreased by the reduction in leaf water potential because of its low *C*_p_ even when the vapour pressure deficit was as low as ~1.5MPa, whereas the higher *g*_s_ in Habataki was supported by the maintenance of higher leaf water potential through the higher hydraulic conductance (Adachi *et al.*, 2011). This is clear because water-stress-relaxation treatments increased *A*_370_ and *g*_s_ in Koshihikari leaves to the same level as in Habataki at similar LNC but had no effect in Habataki leaves (Adachi *et al.*, 2011). This means that rice *g*_s_ is affected markedly by *C*_p_ (Taylaran *et al.*, 2011). Because the NILs in this research were derived from the cross between Koshihikari and Habataki and the measurement conditions of both *A*_370_ and *C*_p_ were exactly the same as in Adachi *et al.* (2011) and Taylaran *et al.* (2011), the connection between *C*_p_ and *g*_s_ may be the case with the rice lines in this research. The combination of NIL(*qCAR4*) and NIL(*qCAR8*) may have resulted in the higher *C*_p_ in NIL(*qCAR4*+*qCAR8*) than in Koshihikari (Fig. 3A) and thus in the high *g*_s_. The even greater *C*_p_ in Habataki might thus explain the far larger *g*_s_ than that of NIL(*qCAR4*+*qCAR8*). These assumptions should be tested by further water-stress-relaxation experiments.

The high *C*_p_ in NIL(*qCAR4*) is attributable to the high *S*_r_, whereas the high *C*_p_ in NIL(*qCAR8*) is attributable to the high *L*_p_ (Fig. 3B, C). The higher *C*_p_ in NIL(*qCAR4*+*qCAR8*) seems to result from the combination of both. This result also suggests that it is possible to improve *S*_r_ and *L*_p_ simultaneously in rice breeding. *C*_p_ was lower in NIL(*qCAR4*) than in Habataki on account of its smaller *S*_r_. This suggests that other QTLs are associated with the difference in *S*_r_ between Koshihikari and Habataki. Although *L*_p_ in Habataki was somewhat lower than that in Koshihikari, *L*_p_ in NIL(*qCAR8*) was even higher than that in Koshihikari, which seems to be a result of the combination of the Habataki chromosome segment with the Koshihikari background. *L*_p_ is controlled by the activity of aquaporins, the water-channel proteins of the cell membrane (Murai-Hatano *et al.*, 2008; Sakurai-Ishikawa *et al.*, 2011), but no open reading frame encoding an aquaporin is found in the region of *qCAR8* (http://rapdb.dna.affrc.go.jp/). Isolating genes underlying *qCAR4* and *qCAR8* will provide useful information on how *S*_r_ and *L*_p_ are controlled in rice plants.

In addition to the high LNC and *g*_s_ in NIL(*qCAR4*+*qCAR8*), another factor may be associated with the high *A*_370_ in NIL(*qCAR4*+*qCAR8*) and might be inherited from NIL(*qCAR4*) (Fig. 4). With reference to the processes that control photosynthesis as mentioned above, this factor is likely to be *g*_m_. Generally, leaves with high LMA show high *g*_m_ owing to the high total chloroplast surface area exposed to intercellular airspaces per leaf area (Hanba *et al.*, 1999; Terashima *et al.*, 2001; Montpied *et al.*, 2009). LMA was significantly higher in NIL(*qCAR4*) and NIL(*qCAR4*+*qCAR8*) than in Koshihikari (Table 1). This result supports the possibility that the high *g*_m_ of each NIL is associated with the high value of *A*_370_. The result that LMA in Habataki did not differ from that in Koshihikari suggests that the high LMA in NIL(*qCAR4*) and NIL(*qCAR4*+*qCAR8*) is a result of the combination of the Habataki chromosome segment with the Koshihikari background. It is known that LMA is also related to LNC (Niinemets *et al.*, 1999). However, the correlation coefficient between these two parameters in this study was not high (0.53 for paddy and 0.47 for pots), suggesting that the LNC was primarily controlled by factors other than LMA. This study cannot rule out the possibility that differences in the Michaelis–Menten constant, the maximum carboxylation rate, and the activation state of Rubisco are associated with the difference in *A*_370_ (Ishikawa *et al.*, 2011), although these traits seem to be similar among rice cultivars (Makino *et al.*, 1987). Further studies should clarify which traits are related to the high *A*_370_ in NIL(*qCAR4*) and NIL(*qCAR4*+*qCAR8*).

The flowering time of NIL(*qCAR8*) and NIL(*qCAR4*+*qCAR8*) was 7 days earlier than that of Koshihikari, while that of NIL(*qCAR4*) was similar to that of Koshihikari. This indicates that the genetic region of *qCAR8* includes genes associated with flowering time. The causal link between flowering and photosynthesis is unknown at this time and should be examined in future research.

### Potential for further enhancement of photosynthesis beyond Habataki

Despite having only two out of the four QTLs, NIL(*qCAR4*+*qCAR8*) had the same *A*_370_ as Habataki. This phenomenon may be explained by the combined effect of the two Habataki chromosome segments in the Koshihikari genetic background. The process of photosynthesis is complex and is controlled by many genes (Shi *et al.*, 2005). Its improvement might be a challenging task (Flood *et al.*, 2011). However, the current results suggest that it is possible to increase *P*_n_ to the highest level known by introducing a small number of genes from donor plants if the right combinations of cultivars are selected. It might be possible to develop a rice with a *P*_n_ even higher than that of Habataki if the additional QTLs for *P*_n_ on chromosomes 5 and 11 (Fig. 2) were stacked into NIL(*qCAR4*+*qCAR8*). To test this hypothesis, this study group is developing rice lines that carry multiple QTLs for *P*_n_ in the genetic background of Koshihikari.

### Conclusion

The *P*_n_ of NIL(*qCAR4*+*qCAR8*) was as high as that of Habataki, even though the NIL had only two of the four known QTLs for *P*_n_. The high values of LNC, *S*_r_, and possibly *g*_m_ found in NIL(*qCAR4*) and of *L*_p_ in NIL(*qCAR8*) combined in NIL(*qCAR4*+*qCAR8*) to increase *P*_n_. The increases in some of these traits cannot be explained in isolation from the Koshihikari genetic background. These results suggest that QTL pyramiding is a powerful approach in breeding of rice for increased *P*_n_, and it should be possible to develop rice with even higher capacity for photosynthesis by stacking other QTLs into NIL(*qCAR4*+*qCAR8*).

## Supplementary material

Supplementary data are available at *JXB* online.

Supplementary Fig. S1. Relationships between leaf N content and stomatal conductance in flag leaves.

Supplementary Data
