# Supplementary material for: Introgression of two chromosome regions for leaf photosynthesis from an indica rice into the genetic background of a japonica rice
Source: J Exp Bot. 2014 Mar 3;65(8):2049–56. doi: 10.1093/jxb/eru047 (PMC3991736; doi:10.1093/jxb/eru047)
Supplement: Supplementary Data [file supp_65_8_2049__index.html]

Introgression of two chromosome regions for leaf photosynthesis from an indica rice into the genetic background of a japonica rice — Introgression of two chromosome regions for leaf photosynthesis from an indica rice into the genetic background of a japonica rice — Supplementary Data 

# Introgression of two chromosome regions for leaf photosynthesis from an *indica* rice into the genetic background of a *japonica* rice

## Supplementary Data

Data files

**Files in this Data Supplement:**

- Supplementary Data - Supplementary Data
