# Supplementary material for: Introgression of two chromosome regions for leaf photosynthesis from an indica rice into the genetic background of a japonica rice
Source: J Exp Bot. 2014 Mar 3;65(8):2049–56. doi: 10.1093/jxb/eru047 (PMC3991736; doi:10.1093/jxb/eru047)
Supplement: Supplementary Data [file supp_eru047_jexbot108787_file001.pdf]

Supplementary data

Introgression of two chromosome regions for leaf photosynthesis from an *indica* rice into the genetic background of a *japonica* rice

Shunsuke Adachi, Leticia Z. Baptista, Tomohiro Sueyoshi, Kazumasa Murata, Toshio Yamamoto, Takeshi Ebitani, Taiichiro Ookawa, Tadashi Hirasawa

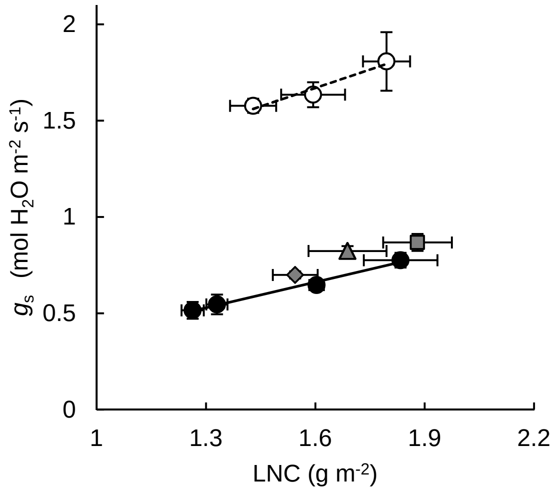

Fig. S1. Relationship between leaf N content (LNC) and stomatal conductance (g_s) at a PPFD of 2000 µmol m^{-2} s^{-1} and an ambient CO_2 concentration of 370 µmol mol^{-1} in flag leaves of Koshihikari (filled circles), Habataki (open circles), NIL(*qCAR4*) (triangle), NIL(*qCAR8*) (diamond) and NIL(*qCAR4*+*qCAR8*) (square) grown in 12 litre pots. The solid and broken lines indicate the regression line for Koshihikari and Habataki, respectively. Error bars indicate the SD for n = 4–6.
